# Taking stock of most-favored nation deals: how have markets reacted?

**DOI:** 10.1093/haschl/qxag176

**Published:** 2026-07-09

**Authors:** Noam Y Kirson, John M Dooley, Zhiqing Ge, Amitabh Chandra

**Affiliations:** Analysis Group, Inc., Boston, MA 02199, USA; Analysis Group, Inc., Dallas, TX 75219, USA; Analysis Group, Inc., Dallas, TX 75219, USA; Harvard Kennedy School, Harvard University, Cambridge, MA 02138, USA; Harvard Business School, Harvard University, Boston, MA 02163, USA

**Keywords:** most-favored nation, drug pricing, event study

## Abstract

**Introduction:**

The Trump Administration has pursued most-favored nation (MFN) drug pricing agreements with major pharmaceutical manufacturers, raising concerns about potential effects on pharmaceutical revenues, profitability, and innovation incentives. We examined whether equity markets interpreted these developments as positive or negative for the pharmaceutical sector.

**Methods:**

We conducted an event study of 16 publicly traded pharmaceutical firms announcing MFN agreements. Abnormal returns and cumulative abnormal returns were estimated using standard market-model regressions based on each firm's historical relationship to the S&P 500. We analyzed market reactions surrounding “Liberation Day,” the date MFN letters were announced, and subsequent agreement announcements.

**Results:**

The strongest market response was observed following the first MFN agreement announced by Pfizer, with largely positive market reactions across most of the pharmaceutical firms analyzed. In contrast, “Liberation Day” generated limited market reaction, while the MFN letters produced more modest negative effects.

**Conclusion:**

Investors did not interpret the announced MFN agreements as materially value destructive, likely because the deals primarily targeted Medicaid and the limited TrumpRx platform, while also resolving some regulatory and tariff uncertainty. Future expansion of MFN may lead to different market reactions.

Key Takeaways

- Using an event study methodology across 16 pharmaceutical firms, we find that most-favored nation (MFN) deals announced to date have not been perceived by market investors as value destructive.
- Our findings suggest that markets viewed the negotiated deals as substantially less harmful than feared while likely resolving regulatory and tariff uncertainty.
- However, the deals so far have focused on Medicaid and the more limited TrumpRx channel; future expansion of MFN may lead to substantially different market reactions.

## Introduction

On July 31, 2025, the Trump Administration sent letters to 17 large pharmaceutical manufacturers outlining steps to implement most-favored nation (MFN) drug pricing in the United States, under which American patients would pay no more for a drug than patients in other wealthy countries. With the recent announcement by Regeneron, all 17 firms have now announced specific MFN pricing agreements with the Administration. In practice, the agreements announced to date have focused primarily on Medicaid, a channel that already carries deep discounts, as well as TrumpRx, a fairly narrow government-run platform that connects consumers to direct-purchase programs or coupon-based discounts. Available information suggests that the agreements included additional dimensions. For example, the agreements also offered companies some protection from tariff risk, introduced by the Administration's “Liberation Day” announcement, in exchange for commitments to invest in US-based manufacturing.

The central question we ask is simple: did investors view these MFN agreements as good news or bad news for the pharmaceutical industry? This matters because policies intended to lower drug prices have historically raised investor concern, given their potential to compress revenues and profit margins, and ultimately to reduce incentives for pharmaceutical innovation. Stock markets are a natural lens through which to answer this question, since share prices reflect investors’ collective judgment about a company's expected future profitability. We examined how markets responded to MFN-related announcements using an event study methodology.

## Methods and data

To assess whether investors viewed MFN agreements as good news or bad news, we use a standard tool in financial economics called an event study.^1^ The core idea is straightforward: On any given day, a company's stock price moves partly because of broad market forces (the overall stock market rising or falling) and partly because of news specific to that company. An event study separates these two sources of movement. It measures how much the stock's actual movement deviated from its expected movement (based on the market conditions) around the time of a specific announcement. A positive deviation means investors updated upward their assessment of the company's expected future profits; a negative deviation means they updated downward. By measuring these deviations around key MFN-related dates, we can draw direct inferences about how investors interpreted each announcement.

Using the Refinitiv Datastream from the London Stock Exchange Group Workspace, we collected US stock price data for the 16 public companies that have announced MFN deals with the Administration. Boehringer Ingelheim, a private company, was not included in the event study. For each company, we estimated the abnormal returns (ARs) associated with each announcement date. As an additional check, we also examined a longer event window, calculating the cumulative abnormal returns (CARs) over the day of the announcement and the following day. To estimate abnormal returns, we first estimated the relationship between each company’s returns and the S&P 500 index returns from April 2, 2024, through April 1, 2025, using ordinary least squares regression. The abnormal return is the actual company return less the predicted return from this regression. We conducted several sensitivity analyses. First, we repeated the event study including the Nasdaq Biotechnology Index as an additional control. Second, we conducted the analysis using the Fama–French 5-factor model.^2^ Neither of these alternate specifications materially changed our findings or conclusions. In addition, as several event dates contain multiple firm announcements, introducing potential cross-sectional correlation across firms, we also calculate ARs and CARs for an equal-weighted portfolio consisting of all 16 firms.

Crucially, rather than focusing only on the MFN deal announcements themselves, we also examine market reactions around “Liberation Day” and the date on which MFN letters were first sent to companies. This broader lens allows us to assess the full arc of investor sentiment, from the initial perception of threat to the resolution of uncertainty, and to determine whether any gains at the deal announcement stage simply reflected a recovery of value lost at earlier dates.

## Results

**Figure 1.**
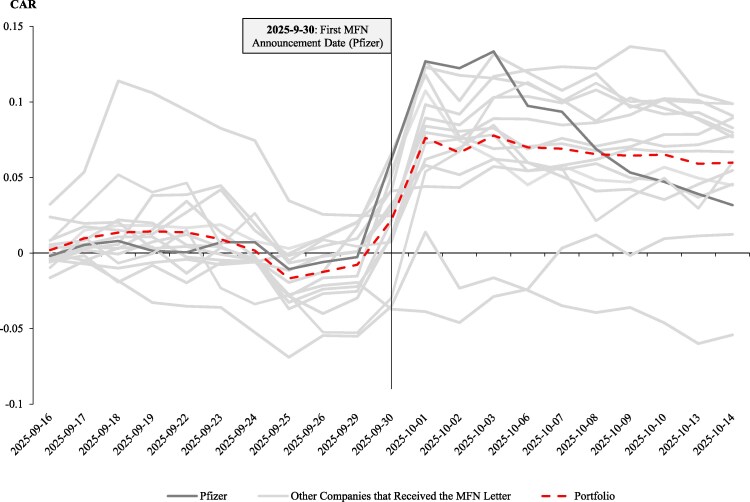
Company and portfolio cumulative abnormal returns (CARs) around the first most-favored nation (MFN) announcement date (Pfizer), 9/16/2025–10/14/2025. Company CARs in the ten trading days prior to Pfizer's MFN announcement were generally small. Company CARs generally increased and remained elevated for the ten trading days after Pfizer's MFN announcement. Source/Notes: SOURCE: Authors’ analysis based on data from London Stock Exchange Group Workspace (Refinitiv Datastream), accessed May 2026**)**. NOTE: Portfolio return is calculated as the simple (equal-weighted) average of the cumulative abnormal returns of all companies in the sample.

The biggest market mover was the first formal agreement announcement by Pfizer. Figure 1 depicts the CARs for all 16 companies, as well as the average across them. On the Pfizer announcement day, ARs for 9 of the 16 firms were positive and significant (*P* < 0.05), and 2 more were borderline significant (*P* < 0.1). The largest 1-day increases were observed for Merck (6.70%) and Pfizer (6.59%), followed by GSK (4.89%) and Eli Lilly (4.65%). When considering a 2-day event window, we see even larger CARs. The CARs for 14 of the 16 firms were positive and statistically significant (*P* < 0.05), led by Merck (13.93%), Pfizer (13.15%), and Eli Lilly (12.31%). The average CAR over the broader window was 8.5%. We did not find news other than Pfizer's announcement that would explain these returns. Markets clearly saw the details of the Pfizer announcement as a net positive and inferred that future deals for the other companies would follow a similar structure. We indeed find that subsequent deal announcements provided more limited additional information and were largely associated with statistically insignificant ARs, though we observe a handful of both positive and negative movements that vary depending on the length of the event window considered. The detailed company by event-level estimated announcement day ARs in our base-case specification (relative to S&P 500) can be seen in Table 1. Table S1 reports our company-level estimates from the sensitivity analysis, including control for the Nasdaq Biotechnology Index, Table S2 reports the estimates using the Fama–French 5-factor model, and 2-day CARs are provided in Table S3 (relative to S&P 500).

**Table 1.** Abnormal returns on liberation day and key MFN-related events relative to the S&P 500 index.

			Liberation Day	MFN letter date	MFN announcement dates							
	Company/portfolio	Stock ticker	4/2/2025	7/31/2025	9/30/2025	10/10/2025	10/16/2025	11/6/2025	12/19/2025	1/8/2026	1/12/2026	4/23/2026
[1]	Pfizer	PFE	0.56%	−2.05%	6.59%***	−1.11%	−0.43%	1.31%	0.46%	0.11%	−0.79%	−0.31%
[2]	AstraZeneca	AZN	−0.41%	−5.46%**	2.38%	**−0**.**51%**	0.61%	2.18%^a^	0.50%	−1.16%	−2.87%	−1.30%
[3]	EMD Serono	MKKGY	0.29%	−1.55%	3.22%**	0.44%	−4.67%***	0.79%	−0.39%	−0.23%	2.60%*	−3.96%***
[4]	Eli Lilly	LLY	1.16%	−2.37%	4.65%***	−0.60%	−0.38%	**2**.**09%**	0.79%	−2.05%	1.55%	−0.08%
[5]	Novo Nordisk	NVO	0.12%	−5.63%**	−0.04%	−1.09%	−0.49%	**−3**.**17%**	0.69%	1.58%	1.90%	−1.12%
[6]	Amgen	AMGN	−0.73%	−1.97%	2.74%*	−0.61%	−0.25%	−0.87%	**0**.**48%**	−3.46%**	−0.27%	0.94%
[7]	BMS	BMY	0.07%	−6.06%***^a^	2.10%	−1.84%	−0.60%	0.22%	**1**.**60%**	−1.51%	−0.21%	0.29%
[8]	Genentech	RHHBY	−4.49%***	−2.89%**	4.13%***	0.89%	0.52%	1.49%	**1**.**54%**	−0.66%	−0.49%	1.32%^a^
[9]	Gilead	GILD	0.30%	−2.32%	−1.67%	0.47%	−0.50%	0.60%	**2**.**03%**	−3.18%**	1.04%	0.42%
[10]	GSK	GSK	−0.64%	−4.69%***	4.89%***	0.62%	0.11%	1.06%	**0**.**61%**	−0.74%	0.03%	−0.02%
[11]	Merck	MRK	−0.47%	−4.50%***	6.70%***	−1.92%	−0.21%	1.60%	**0**.**55%**	2.25%*	−1.13%	1.55%
[12]	Novartis	NVS	0.95%	−2.57%**	3.19%***	−0.77%	0.56%	0.81%	**0**.**47%**	−0.45%	0.11%	0.05%
[13]	Sanofi	SNY	−0.73%	−7.58%***^a^	3.33%**	−1.36%	2.19%	1.06%	**0**.**36%**	−0.48%	−2.95%**	1.37%^a^
[14]	Johnson & Johnson	JNJ	1.45%	−1.57%	2.12%*	−0.54%	0.41%	0.38%	−0.83%	**−0**.**84%**	2.59%**	1.94%*
[15]	AbbVie	ABBV	−0.64%	−0.13%^a^	3.60%**	0.26%	0.35%	1.17%	1.64%	−4.09%***	**−0**.**07%**	0.25%
[16]	Regeneron	REGN	1.29%	−1.30%	0.29%	0.45%	−0.79%	1.10%	2.36%	−1.20%	−3.52%**	2.95%**
[17]	Portfolio^b^	—	−0.14%	−3.30%***	3.00%***	−0.44%	−0.23%	0.73%	0.78%	−1.02%	−0.17%	0.26%

NOTES: For each company, the relationship between its returns and the S&P 500's returns is estimated from April 2, 2024, through April 1, 2025, using ordinary least squares (OLS). Based on the estimated relationship for each company, the expected returns are calculated for each announcement date. The abnormal return (AR) is the actual company return less the predicted return. The t-statistic for each company announcement AR is calculated as the AR divided by the product of the square root of the event window length (1 in this case) and the estimated daily AR standard deviation (ie, the standard deviation of residuals from the OLS estimation). Days on which a company announced earnings were dropped from the estimation window (April 2, 2024-April 1, 2025) for that company. Bolded entries correspond to the announcement date of each company's MFN pricing agreement. “***” indicates statistical significance at the 1% level (*P* < 0.01), “**” indicates statistical significance at the 5% level (*P* < 0.05), and “*” indicates significance at the 10% level (*P* < 0.1).

SOURCE: Authors’ analysis based on data and reported earnings announcement dates obtained from London Stock Exchange Group Workspace (Refinitiv Datastream), accessed May 2026.

Abbreviation: MFN, most-favored nation.

^a^Subject to potential confounding from same-day earnings announcements.

^b^Portfolio ARs and CARs are calculated for an equal-weighted portfolio consisting of all 16 firms.

We initially hypothesized that the positive reaction to the Pfizer deal may simply be the mirror image of negative reactions to “Liberation Day” and the distribution of the MFN letters (ie, companies recovering value lost due to the perceived threat of tariffs and the announcement of intent to implement MFN pricing); however, our findings only partially match that pattern. For almost all companies (as well as the equally weighted portfolio), we find small and statistically insignificant ARs on “Liberation Day,” regardless of specification. The MFN letters’ date is associated with more negative reactions, with 8 of 16 firms and the overall portfolio experiencing statistically significant (*P* < 0.05) declines on the announcement day (though the 2 largest declines—BMS and Sanofi—may be related to earnings announcements on the same day). Interestingly, a 2-day event window shows a more muted effect of the July 31st letters. The overall portfolio effects did not differ materially from individual company estimates, including on dates comprising multi-company events. For many of the companies, their positive AR on Pfizer's announcement date eclipses the combined AR response to “Liberation Day” and the MFN letters. That said, for a handful of companies, depending on the precise empirical specification, we do see some evidence of more pronounced negative effects from the earlier dates that more closely matches the recovery hypothesis.

## Discussion

Our analysis suggests that equity markets did not interpret the MFN agreements announced to date as value destructive for the pharmaceutical sector. The strong positive reaction to the Pfizer announcement is consistent with the resolution of regulatory and tariff uncertainty. The announced deals focused primarily on Medicaid, an already heavily discounted channel, and on the narrow TrumpRx program, limiting the overall revenue exposure of the companies involved.

The market reactions are best understood as a sequence of 3 distinct episodes. First, “Liberation Day” was perceived as a real but manageable cost: investors had already priced in some tariff risk, and pharmaceutical manufacturers were not disproportionately affected (thus almost all ARs were small and not statistically significant). Second, the MFN letters represented a more targeted threat, with the government signaling direct action against specific companies’ revenue streams under conditions of considerable uncertainty about the eventual deal terms. Third, the Pfizer announcement resolved much of that uncertainty. Markets had been bracing for mandatory price controls or unilateral government dictates expected to have significant consequences for profit margins. The negotiated deal structure was likely substantially less damaging than what had been feared.

The announced MFN deals are, however, unlikely to be the end of the story. The Trump Administration continues to pursue additional MFN-related policies, most notably the Global Benchmark for Efficient Drug Pricing and Guarding US Medicare Against Rising Drug Costs proposed rules, which target Medicare Part B and D pricing. These programs cover a far larger and more commercially significant share of drug revenues than Medicaid, and the market consequences of such rules, if implemented, could be substantially larger than what we document here.

## Supplementary Material

qxag176_Supplementary_Data
