# Supplementary material for: Taking stock of most-favored nation deals: how have markets reacted?
Source: Health Aff Sch. 2026 Jul 9;4(7):qxag176. doi: 10.1093/haschl/qxag176 (PMC13404990; doi:10.1093/haschl/qxag176)
Supplement: qxag176_Supplementary_Data [file qxag176_supplementary_data.zip › qxag176_supplementary_data_edited.docx]

**Supplemental Table 1:**

**Abnormal Returns on Liberation Day and Key MFN-Related Events Relative to the S&P 500 and Nasdaq Biotechnology Indexes.**

|  |  |  | Liberation Day | MFN Letter Date | MFN Announcement Dates | | | | | | | |
| --- | --- | --- | --- | --- | --- | --- | --- | --- | --- | --- | --- | --- |
|  | **Company / Portfolio** | **Stock Ticker** | **4/2/2025** | **7/31/2025** | **9/30/2025** | **10/10/2025** | **10/16/2025** | **11/6/2025** | **12/19/2025** | **1/8/2026** | **1/12/2026** | **4/23/2026** |
| [1] | Pfizer | PFE | -0.47% | -1.72% | **6.04%***** | -1.71% | -0.61% | 0.60% | -0.87% | 1.79% | -0.20% | 0.48% |
| [2] | AstraZeneca | AZN | -0.86% | -5.30%** | 2.14% | **-0.77%** | 0.54% | 1.86%⁽^a^⁾ | -0.09% | -0.39% | -2.59% | -0.93% |
| [3] | EMD Serono | MKKGY | -0.30% | -1.35% | 2.91%** | 0.11% | **-4.76%***** | 0.38% | -1.16% | 0.76% | 2.95%** | -3.48%** |
| [4] | Eli Lilly | LLY | 0.73% | -2.22% | 4.42%*** | -0.83% | -0.45% | **1.80%** | 0.23% | -1.33% | 1.81% | 0.27% |
| [5] | Novo Nordisk | NVO | -0.34% | -5.49%** | -0.28% | -1.32% | -0.56% | **-3.47%** | 0.10% | 2.33% | 2.17% | -0.77% |
| [6] | Amgen | AMGN | -1.95% | -1.56% | 2.10% | -1.28% | -0.44% | -1.69% | **-1.09%** | -1.44% | 0.45% | 1.90% |
| [7] | BMS | BMY | -0.77% | -5.78%***⁽^a^⁾ | 1.66% | -2.33% | -0.73% | -0.36% | **0.51%** | -0.10% | 0.30% | 0.97% |
| [8] | Genentech | RHHBY | -5.12%*** | -2.68%** | 3.80%*** | 0.54% | 0.42% | 1.06% | **0.73%** | 0.38% | -0.12% | 1.81%⁽^a^⁾ |
| [9] | Gilead | GILD | -0.69% | -1.97% | -2.18% | -0.06% | -0.64% | -0.06% | **0.74%** | -1.51% | 1.64% | 1.23% |
| [10] | GSK | GSK | -1.34% | -4.46%*** | 4.53%*** | 0.20% | -0.01% | 0.58% | **-0.29%** | 0.43% | 0.45% | 0.53% |
| [11] | Merck | MRK | -1.08% | -4.30%*** | 6.38%*** | -2.26%* | -0.31% | 1.19% | **-0.24%** | 3.26%*** | -0.77% | 2.04%* |
| [12] | Novartis | NVS | 0.35% | -2.37%** | 2.87%*** | -1.10% | 0.47% | 0.41% | **-0.30%** | 0.56% | 0.47% | 0.53% |
| [13] | Sanofi | SNY | -1.51% | -7.33%***⁽^a^⁾ | 2.93%** | -1.79% | 2.06%* | 0.54% | **-0.63%** | 0.80% | -2.50%** | 1.98%⁽^a^⁾ |
| [14] | Johnson & Johnson | JNJ | 0.73% | -1.33% | 1.74%* | -0.95% | 0.30% | -0.12% | -1.76%* | **0.36%** | 3.02%*** | 2.51%** |
| [15] | AbbVie | ABBV | -1.33% | 0.10%⁽^a^⁾ | 3.24%** | -0.13% | 0.23% | 0.70% | 0.75% | -2.95%** | **0.34%** | 0.80% |
| [16] | Regeneron | REGN | 0.05% | -0.90% | -0.36% | -0.24% | -0.99% | 0.26% | 0.76% | 0.84% | -2.79%** | **3.92%***** |
| [17] | Portfolio^(b)^ | - | -0.90% | -3.05%*** | 2.60%*** | -0.87% | -0.35% | 0.22% | -0.20% | 0.25% | 0.29% | 0.86% |

NOTES: For each company, the relationship between its returns and the returns of S&P 500 and Nasdaq Biotechnology Indexes is estimated from April 2, 2024 through April 1, 2025 using ordinary least squares (“OLS”). Based on the estimated relationship for each company, the expected returns are calculated for each announcement date. The abnormal return (“AR”) is the actual company return less the predicted return. The t-statistic for each company announcement AR is calculated as the AR divided by the square root of the event window length (one in this case) multiplied by the estimated daily AR standard deviation (i.e., the standard deviation of residuals from the OLS estimation). Days on which a company announced earnings were dropped from the estimation window (April 2, 2024 through April 1, 2025) for that company. Bolded entries correspond to the announcement date of each company's MFN pricing agreement. “***” indicates statistical significance at the 1% level (p < 0.01), “**” indicates statistical significance at the 5% level (p < 0.05), and “*” indicates significance at the 10% level (p < 0.1).
SOURCE: Authors' analysis based on data and reported earnings announcement dates obtained from London Stock Exchange Group Workspace (Refinitiv Datastream), accessed May 2026.
(a) Subject to potential confounding from same-day earnings announcements.
(b) Portfolio ARs and CARs are calculated for an equal-weighted portfolio consisting of all 16 firms.

**Supplemental Table 2:**

**Abnormal Returns on Liberation Day and Key MFN-Related Events Relative to the Fama-French 5 Factor Model.**

|  |  |  | Liberation Day | MFN Letter Date | MFN Announcement Dates | | | | | | | |
| --- | --- | --- | --- | --- | --- | --- | --- | --- | --- | --- | --- | --- |
|  | **Company / Portfolio** | **Stock Ticker** | **4/2/2025** | **7/31/2025** | **9/30/2025** | **10/10/2025** | **10/16/2025** | **11/6/2025** | **12/19/2025** | **1/8/2026** | **1/12/2026** | **4/23/2026** |
| [1] | Pfizer | PFE | 0.46% | -1.38% | **6.83%***** | -1.03% | 0.33% | 1.06% | 0.82% | -0.34% | -0.68% | -0.67% |
| [2] | AstraZeneca | AZN | -0.65% | -5.82%** | 2.34% | **-0.78%** | 0.25% | 2.82%⁽^a^⁾ | 0.32% | -0.98% | -3.16% | -0.77% |
| [3] | EMD Serono | MKKGY | -0.12% | -1.61% | 3.38%** | 0.23% | **-4.57%***** | 1.39% | -0.40% | -0.33% | 2.34% | -3.54%** |
| [4] | Eli Lilly | LLY | 1.10% | -3.14%* | 4.73%*** | -0.93% | -1.03% | **2.75%*** | -0.40% | -0.22% | 1.35% | 0.98% |
| [5] | Novo Nordisk | NVO | -0.09% | -6.26%*** | 0.01% | -1.21% | -0.88% | **-2.69%** | 0.27% | 2.05% | 1.74% | -0.57% |
| [6] | Amgen | AMGN | -0.86% | -1.56% | 2.82%* | -0.54% | 0.21% | -1.22% | **1.32%** | -4.81%*** | -0.20% | 0.34% |
| [7] | BMS | BMY | -0.13% | -5.36%***⁽^a^⁾ | 2.42% | -1.49% | 0.47% | -0.12% | **2.07%** | -2.27% | 0.00% | -0.28% |
| [8] | Genentech | RHHBY | -4.59%*** | -2.63%* | 4.23%*** | 0.81% | 0.78% | 1.47% | **1.72%** | -0.89% | -0.51% | 1.24%⁽^a^⁾ |
| [9] | Gilead | GILD | 0.11% | -1.98% | -1.42% | 0.73% | 0.17% | 0.50% | **2.15%** | -3.44%** | 1.15% | 0.20% |
| [10] | GSK | GSK | -0.63% | -4.45%*** | 4.93%*** | 0.57% | 0.29% | 0.89% | **0.89%** | -1.12% | 0.07% | -0.24% |
| [11] | Merck | MRK | -0.44% | -4.60%*** | 6.73%*** | -1.73% | -0.10% | 1.38% | **0.61%** | 2.07%* | -0.99% | 1.33% |
| [12] | Novartis | NVS | 1.06% | -2.14%** | 3.18%*** | -0.73% | 0.87% | 0.36% | **1.03%** | -1.19% | 0.24% | -0.48% |
| [13] | Sanofi | SNY | -0.73% | -7.16%***⁽^a^⁾ | 3.43%** | -1.41% | 2.53%* | 0.95% | **0.51%** | -0.57% | -2.93%** | 1.23%⁽^a^⁾ |
| [14] | Johnson & Johnson | JNJ | 1.39% | -0.91% | 2.48%** | -0.08% | 1.53% | -0.20% | -0.48% | **-1.35%** | 2.96%*** | 1.25% |
| [15] | AbbVie | ABBV | -0.44% | 0.41%⁽^a^⁾ | 3.74%** | 0.52% | 0.97% | 0.39% | 2.09% | -4.65%*** | **0.28%** | -0.48% |
| [16] | Regeneron | REGN | 1.20% | -1.26% | 0.17% | 0.20% | -0.98% | 1.10% | 2.97%** | -2.18% | -3.67%** | **2.78%*** |
| [17] | Portfolio^(b)^ | - | -0.22% | -3.10%*** | 3.11%*** | -0.44% | 0.05% | 0.67% | 0.95% | -1.26% | -0.15% | 0.14% |

NOTES: For each company, the relationship between its returns and the Fama-French 5 factor model is estimated from April 2, 2024 through April 1, 2025 using ordinary least squares (“OLS”). Based on the estimated relationship for each company, the expected returns are calculated for each announcement date. The abnormal return (“AR”) is the actual company return less the predicted return. The t-statistic for each company announcement AR is calculated as the AR divided by the square root of the event window length (one in this case) multiplied by the estimated daily AR standard deviation (i.e., the standard deviation of residuals from the OLS estimation). Days on which a company announced earnings were dropped from the estimation window (April 2, 2024 through April 1, 2025) for that company. Bolded entries correspond to the announcement date of each company's MFN pricing agreement. “***” indicates statistical significance at the 1% level (p < 0.01), “**” indicates statistical significance at the 5% level (p < 0.05), and “*” indicates significance at the 10% level (p < 0.1).
SOURCES: Authors' analysis based on data and reported earnings announcement dates obtained from London Stock Exchange Group Workspace (Refinitiv Datastream), accessed May 2026. Fama-French 5 Research Factors (2x3) Daily, available at https://mba.tuck.dartmouth.edu/pages/faculty/ken.french/data_library.html, accessed June 2026.

(a) Subject to potential confounding from same-day earnings announcements.

(b) Portfolio ARs and CARs are calculated for an equal-weighted portfolio consisting of all 16 firms.

**Supplemental Table 3:**

**Cumulative Abnormal Returns around Liberation Day and Key MFN-Related Dates Relative to the S&P 500 Index.**

|  |  |  | Liberation Day | MFN Letter Date | MFN Announcement Dates | | | | | | | |
| --- | --- | --- | --- | --- | --- | --- | --- | --- | --- | --- | --- | --- |
|  | **Company / Portfolio** | **Stock Ticker** | **4/2/2025** | **7/31/2025** | **9/30/2025** | **10/10/2025** | **10/16/2025** | **11/6/2025** | **12/19/2025** | **1/8/2026** | **1/12/2026** | **4/23/2026** |
| [1] | Pfizer | PFE | 0.15% | -0.73% | **13.15%***** | -1.61% | 0.66% | 1.40% | 0.46% | 0.78% | -1.15% | 0.80% |
| [2] | AstraZeneca | AZN | 1.52% | -5.49% | 10.82%*** | **-0.33%** | 1.73% | 0.54%⁽^a^⁾ | 0.33% | 0.24% | -1.15% | -2.52% |
| [3] | EMD Serono | MKKGY | 0.60% | -0.89% | 11.81%*** | 0.42% | **-2.21%** | 0.67% | 0.97% | 0.23% | 0.35% | -3.14% |
| [4] | Eli Lilly | LLY | 1.11% | 1.79% | 12.31%*** | -3.36% | -2.75% | **0.63%** | 0.84% | -4.48%* | 1.38% | -4.34%* |
| [5] | Novo Nordisk | NVO | 1.76% | -2.04% | 6.40%** | -0.82% | -3.69% | **-4.81%** | 0.55% | 3.95% | 1.89% | 5.28% |
| [6] | Amgen | AMGN | 2.82% | -0.67% | 8.18%*** | -0.55% | 0.49% | 0.49% | **1.38%** | -5.01%** | -0.60% | -0.63% |
| [7] | BMS | BMY | -3.31% | -4.11%*⁽^a^⁾ | 7.10%*** | -2.76% | -0.07% | 0.30% | **2.45%** | -1.60% | 0.00% | -0.24% |
| [8] | Genentech | RHHBY | -1.66% | -2.73% | 10.94%*** | -0.41% | 0.85% | 3.16%* | **1.68%** | 0.29% | -0.25% | 0.62%⁽^a^⁾ |
| [9] | Gilead | GILD | 1.07% | -1.54% | -1.75% | 1.01% | 3.40% | -3.36% | **1.68%** | -3.07% | 0.19% | -2.29% |
| [10] | GSK | GSK | 3.59%* | -3.34% | 10.21%*** | 0.82% | 0.42% | 0.10% | **0.54%** | -0.43% | -0.87% | -2.23% |
| [11] | Merck | MRK | -1.09% | -3.09%* | 13.93%*** | -2.04% | 0.94% | 2.27% | **4.21%**** | 1.96% | -1.94% | -0.70% |
| [12] | Novartis | NVS | 2.39% | -1.23% | 5.78%*** | -1.61% | 0.96% | 0.56% | **0.96%** | -0.49% | -0.11% | -1.43% |
| [13] | Sanofi | SNY | 0.77% | -5.09%***⁽^a^⁾ | 8.61%*** | 0.04% | 3.36%* | 1.41% | **0.03%** | 1.35% | -3.78%** | -0.25%⁽^a^⁾ |
| [14] | Johnson & Johnson | JNJ | 3.65%** | -0.21% | 2.50% | -0.25% | 1.05% | 0.18% | -0.29% | **-1.43%** | 4.42%*** | 0.66% |
| [15] | AbbVie | ABBV | -1.75% | 3.29%⁽^a^⁾ | 8.93%*** | -0.06% | 1.43% | 1.18% | 2.00% | -6.02%*** | **0.26%** | -1.00% |
| [16] | Regeneron | REGN | 1.22% | 2.02%⁽^a^⁾ | 6.82%*** | -1.25% | 0.73% | 2.88% | 3.75%* | -1.84% | -4.43%** | **0.90%** |
| [17] | Portfolio^(b)^ | - | 0.80% | -1.52% | 8.45%*** | -0.82% | 0.43% | 0.45% | 1.31% | -1.01% | -0.39% | -0.69% |

NOTES: For each company, the relationship between its returns and the S&P 500's returns is estimated from April 2, 2024 through April 1, 2025 using ordinary least squares (“OLS”). Based on the estimated relationship for each company, the expected returns are calculated for the two trading days on and subsequent to each announcement date (0, +1). The abnormal return (“AR”) is the actual company return less the predicted return. The cumulative abnormal return (“CAR”) is the sum of the abnormal returns over the two-day event window. The t-statistic for each company announcement CAR is calculated as the CAR divided by the square root of the event window length (two in this case) multiplied by the estimated daily AR standard deviation (i.e., the standard deviation of residuals from the OLS estimation). Days on which a company announced earnings were dropped from the estimation window (April 2, 2024 through April 1, 2025) for that company. Bolded entries correspond to the announcement date of each company's MFN pricing agreement. “***” indicates statistical significance at the 1% level (p < 0.01), “**” indicates statistical significance at the 5% level (p < 0.05), and “*” indicates significance at the 10% level (p < 0.1).

SOURCE: Authors' analysis based on data and reported earnings announcement dates obtained from London Stock Exchange Group Workspace (Refinitiv Datastream), accessed May 2026.

(a) Subject to potential confounding from earnings announcements occurring within the calculation window (0, +1).

(b) Portfolio ARs and CARs are calculated for an equal-weighted portfolio consisting of all 16 firms.
